# Bilateral vocal fold injection with autologous fat in patients with vocal fold atrophy with or without sulcus

**DOI:** 10.1007/s00405-019-05479-5

**Published:** 2019-05-27

**Authors:** Emke M. J. M. van den Broek, Bas J. Heijnen, Martine Hendriksma, Vivienne A. H. van de Kamp-Lam, Antonius P. M. Langeveld, Peter Paul G. van Benthem, Elisabeth V. Sjögren

**Affiliations:** 1 Department of Otorhinolaryngology/Head and Neck Surgery, Leiden University Medical Centre, Albinusdreef 2, PO-box 9600, 2300 RC Leiden, The Netherlands; 2 Department of Otorhinolaryngology/Head and Neck Surgery, University Medical Centre, Utrecht, The Netherlands

**Keywords:** Glottic insufficiency, Vocal fold atrophy, Sulcus, Autologous fat, Vocal fold injection

## Abstract

**Purpose:**

To evaluate voice outcome after bilateral vocal fold injection with autologous fat in patients with non-paralytic glottic insufficiency due to vocal fold atrophy with or without sulcus.

**Methods:**

This is a retrospective cohort study from September 2012 to December 2017 including 23 patients undergoing bilateral vocal fold injection with autologous fat (24 procedures) for vocal fold atrophy (15 procedures) or atrophy with sulcus (Ford type II or III) (9 procedures). Voice data were collected and analyzed for the preoperative and the 3- and 12-month postoperative time points according to a standardized protocol, including Voice Handicap Index (VHI)-30 and perceptive, acoustic and aerodynamic parameters. Failure rate was defined as non-relevant improvement (< 10 points) in VHI-30 at 12 months and number of revisions within 12 months.

**Results:**

There was a clinically relevant (≥ 15 points) and statistically significant improvement in the VHI-30 (preoperative: 49.1 points; postoperative at 12 months: 29.7 points). Change in dynamic range was also statistically significant over time (*p* = 0.028). There were no differences in voice parameters between patients with atrophy only and atrophy with sulcus, although grade tended to be lower in patients with atrophy only over all time points.

**Conclusion:**

This study shows that bilateral vocal fold injection with autologous fat is a beneficial treatment not only for patients with atrophy but also for patients with sulcus. A comparison of the results with those reported from other forms of sulcus surgery confirmed this finding. However, there is a need for further prospective studies comparing the short- and long-term effects of different techniques.

## Introduction

Non-paralytic glottic insufficiency is a common cause of dysphonia. There are several underlying causes, including vocal fold atrophy. In our clinic, we routinely encounter three forms of vocal fold atrophy: vocal fold atrophy in presbyphonia, an adolescent form, and atrophy associated with congenital vocal fold scar in the form of sulcus [1]. If a sulcus is present it can be further classified as a physiologic sulcus (Ford type I) or pathologic sulcus vocalis (Ford types II and III) with Ford types II and III corresponding to a sulcus vergeture and a sulcus vocalis in the classification by Bouchayer and Cornut [2, 3].

The main surgical treatment for atrophy without sulcus is vocal fold medialization, which can be achieved either by bilateral vocal fold injection (VFI) with a durable injectable such as autologous fat or calcium hydroxyapatite, or by bilateral medialization thyroplasty. For vocal fold atrophy with sulcus, several surgical techniques are used that are broadly divided into phonosurgical epithelium freeing techniques such as microflap formation, hydrodissection, angiolytic laser treatment and tissue engineering techniques on the one hand, and medialization techniques on the other. In their consensus report on vocal fold scar, the European Laryngological Society (ELS) considered medialization to be the least traumatizing procedure to the vocal fold and, therefore, suggested that it be used as the initial treatment for vocal fold scar, including sulcus [4]. However, it is also known that the results of medialization for vocal fold atrophy with scar, including sulcus, are less predictable than the results for glottic insufficiencies caused by atrophy alone, hypomobility, or paresis [5]. In this study, we evaluated the prospectively collected voice outcome data after bilateral VFI with autologous fat in patients with vocal fold atrophy with or without sulcus and compared our findings with those reported in the literature.

## Methods

### Patients

**Fig. 1:**
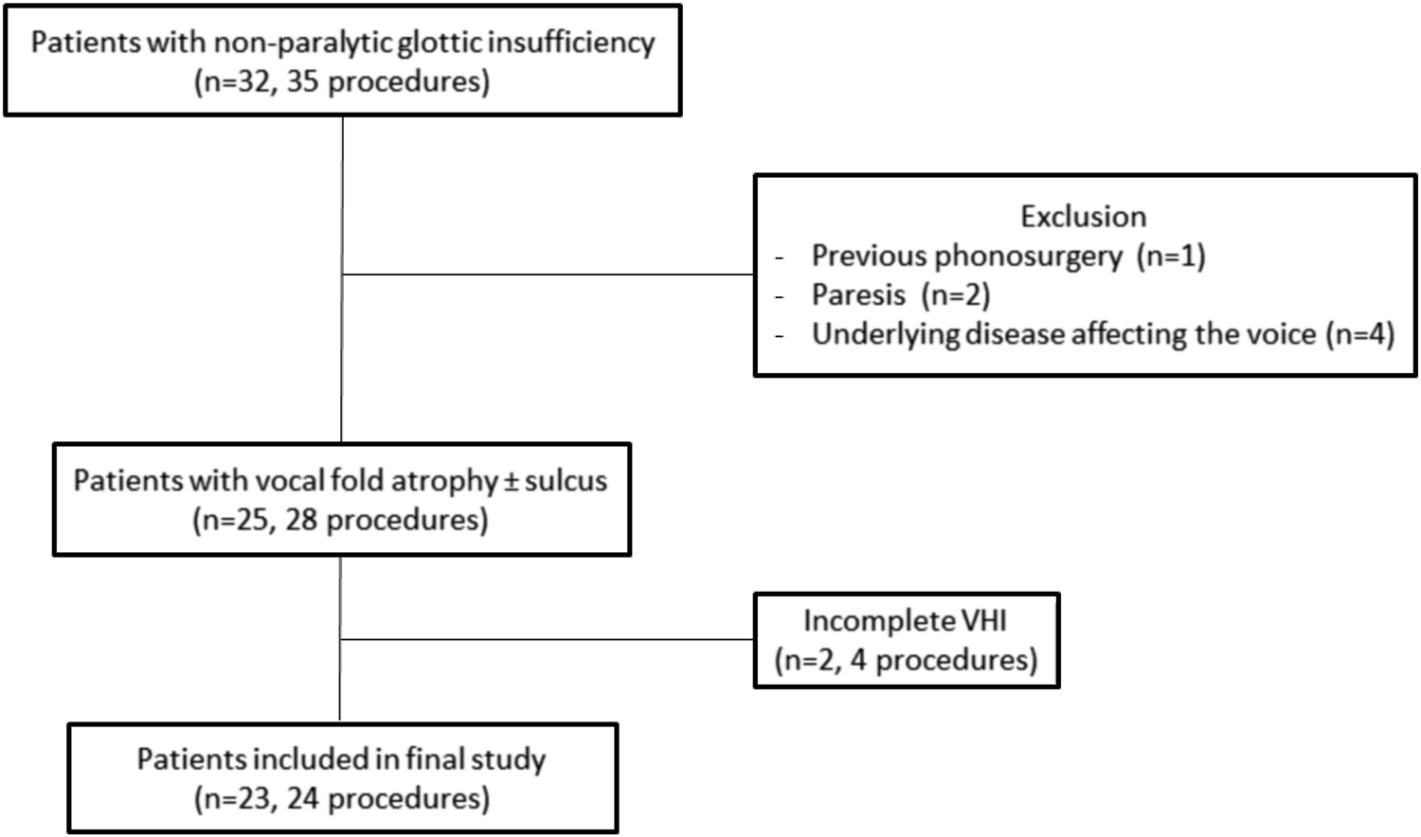
Patient selection and inclusion and exclusion criteria

This study was approved by the Leiden University Medical Centre Ethics Committee. All patients with non-paralytic glottic insufficiency who underwent bilateral VFI with autologous fat under general anesthesia (*n* = 32, procedures = 35) from September 2011 to December 2017 were retrospectively reviewed. Seven patients were excluded because of previous phonosurgery for sulcus (*n* = 1), paresis as another cause of glottic insufficiency (*n* = 2), or an underlying disease affecting the voice (*n* = 4) including Parkinson’s disease (*n* = 2), laryngeal dystonia (*n* = 1), and laryngeal papillomatosis (*n* = 1). Of the 25 remaining patients (28 procedures), 23 (24 procedures) had pre- and postoperative voice data with at least a complete Voice Handicap Index (VHI)-30 questionnaire and were included in the definitive analysis (Fig. 1). These patients had undergone bilateral VFI with autologous fat between September 2012 and December 2017.

### Voice data

Voice outcome data were collected according to a standardized voice analysis protocol implemented preoperatively and at 3 and 12 months postoperatively. This protocol included patients’ self-assessments using the VHI-30, perceptual evaluation using the overall grade score of the GRBAS (Grade, Roughness, Breathiness, Asthenia, Strain) scale, aerodynamic evaluation with maximum phonation time (MPT) and dynamic range, and acoustic analyses including fundamental frequency (F0) and melodic range. The VHI-30 was the primary outcome parameter of the voice analysis protocol. It is a patient-based self-assessment tool consisting of 30 items, which are distributed over three domains: functional, physical, and emotional [6]. In the Dutch version of the VHI-30, a score of 15 points or more identifies patients with voice problems in daily life [7, 8]. Furthermore, a change in pre- and post-operative score of 10 points or more in the individual patient and 15 points or more for a group can be considered clinically relevant [8]. The voice was perceptually graded using the grade of the GRBAS scale ranging from zero to three [9]. Running speech samples in random order were graded by experienced listeners (two senior speech language therapists and one laryngologist) and a consensus was reached through (re)evaluation and discussion. The MPT was measured on /a/ at a comfortable pitch and loudness. The longest MPT from two attempts was included in the analysis. The fundamental frequency in hertz (Hz), dynamic range in decibels (dB), and melodic range in semitones (ST) were extracted from the patient’s phonetogram recorded with the voice profiler (Alphatron, Rotterdam, the Netherlands, 2007) using standardized settings.

### Procedure

All procedures were performed under general anesthesia by an experienced laryngologist and/or a fellowship-trained laryngologist. Bilateral VFI was performed with autologous fat, harvested by abdominal liposuction. In a minority of patients using anticoagulants, a periumbilical incision was made to ensure hemostasis, and fat lobules were harvested and separated from the underlying connective tissue. Subsequently, the fat was centrifuged and separated from blood and its liquid component. The standard practice was injection of fat lateral in the vocal fold (thyroarytenoid muscle) at a level just anterior of the vocal fold process using a Brünings syringe until medialization was achieved, with an anticipated overcorrection of between 1/3 and 1/4 of the vocal fold width at the free edge. The final amount on overcorrection was based on the clinical experience of the surgeon. In some cases, a second injection point at the level of the midcord was needed to obtain the intended result. All patients had absolute voice rest for 24 h after the procedure. Subsequently, they received voice therapy by an experienced speech–language therapist, starting within the first week postoperatively, including resonant voice therapy and vocal hygiene advice.

### Statistical analysis

All data were analyzed using SPSS (IBM SPSS Statistics for Windows, Version 21.0, released 2012. IBM Corp, Armonk, NY, USA). Demographic details were presented as the mean with standard deviation (SD) or as proportions using percentages. The effect of time on the different voice parameters was assessed with the linear mixed model and was adjusted for diagnosis (atrophy versus atrophy with sulcus). The linear mixed model was chosen because it applies a correction for missing data. This correction is based on the observed data and uses all available data, without the need to censure a patients’ entire data, when one or more data points are missing or the need for imputation of measurements [10]. For all statistical tests, a *p* value < 0.05 was considered significant.

## Results

Table 1 shows the preoperative demographic details of the 23 patients undergoing 24 procedures. These were all female patients. Fifteen injections were performed in patients with vocal fold atrophy, two of these were performed consecutively in the same patient within 5 months. Nine injections were performed in patients with atrophy and sulcus (Ford type II or III). The pre- and post-VFI voice outcome data for the overall patient group are shown in Table 2. The changes in VHI-30 (*p* < 0.001) and dynamic range (*p* = 0.028) were statistically significant over time. The change in the VHI-30 from baseline to 12 months postoperation was also clinically relevant (Δ 19.4). The improvement in postoperative outcomes in the other voice parameters was not statistically significant (Table 2).

**Table 1:** Demographic details of the patients undergoing bilateral vocal fold injection with autologous fat

Characteristics	Total = 24 (100%)
Mean age, years at baseline (SD)	39.5 (18.2)
Gender (%)	
Female	24 (100)
Etiology (%)	
Atrophy	15 (62.5)
Atrophy with sulcus	9 (37.5)

*SD* standard deviation

**Table 2:** Pre- and postoperative voice outcome data of patients with vocal fold atrophy ± sulcus undergoing bilateral vocal fold injection with autologous fat

	Preoperative Mean (95% CI)	3 months postoperation Mean (95% CI)	12 months postoperation Mean (95% CI)	*p* value
VHI-30	49.1 (41.4; 56.8)	34.8 (27.0; 42.6)	29.7 (21.5;37.9)	< 0.001*
Grade	1.58 (1.3; 1.9)	1.20 (0.9; 1.5)	1.18 (0.8; 1.6)	0.071
MPT (s)	11.0 (9.2; 12.9)	12.4 (10.5; 14.3)	12.2 (10.0; 14.4)	0.384
Dynamic range (dB)	32.3 (27.5; 37.0)	33.8 (29.1; 38.5)	41.1 (35.4; 46.8)	0.028*
F0 (Hz)	198 (187; 209)	201 (190; 212)	196 (184; 209)	0.730
Melodic range (ST)	17.0 (14.1; 19.8)	17.4 (14.5; 20.3)	19.7 (16.2; 23.2)	0.428

*CI* confidence interval, *VHI* Voice Handicap Index, *MPT* maximum phonation time, *F0* fundamental frequency, *Hz* hertz, *dB* decibels, *ST* semitones

*p value < 0.05 was considered significant

Table 3 shows the results stratified for patients with vocal fold atrophy only and patients with vocal fold atrophy and sulcus. The overall change in the VHI-30 was statistically significant in both groups (*p* < 0.001, *p* = 0.002). The change in the VHI from the preoperative time point to the 12-month postoperative time point was clinically relevant in both groups (Δ 21.2 atrophy; Δ 17.7 sulcus). There was no significant improvement in any of the other voice parameters for the two groups. Finally, there was no significant difference between the two groups in the severity of the scores for the various voice parameters, although the grade of dysphonia tended to be lower in patients with atrophy (mean difference over all time points 0.47, *p* = 0.057, data not shown in table).

**Table 3:** Pre- and postoperative voice outcome data stratified for patients with vocal fold atrophy and vocal fold atrophy with sulcus

	Etiology	Preoperative Mean	3 months postoperation Mean	12 months postoperation Mean	*p* value
VHI-30	Atrophy	47.5	33.0	26.3	< 0.001*
	Sulcus	51.8	38.0	34.1	0.002*
Grade	Atrophy	1.5	0.9	1.0	0.078
	Sulcus	1.8	1.6	1.5	0.602
MPT (s)	Atrophy	11.2	13.4	12.4	0.292
	Sulcus	10.7	10.9	11.8	0.825
Dynamic range (dB)	Atrophy	30.6	33.3	41.6	0.051
	Sulcus	35.0	34.6	40.6	0.449
F0 (Hz)	Atrophy	191	199	194	0.513
	Sulcus	210	205	200	0.642
Melodic range (ST)	Atrophy	15.7	17.5	19.8	0.363
	Sulcus	19.1	17.2	19.6	0.743

*VHI* Voice Handicap Index, *MPT* maximum phonation time, *F0* fundamental frequency, *Hz* hertz, *dB* decibels, *ST* semitones

*p* value < 0.05 was considered significant

Looking at individual procedures, 12-month data were available in 18 out of 24 procedures. Six procedures did not have 12-month data: in four procedures, the patients (*n* = 4) had already undergone or opted for a revision procedure [repeat VFI with fat (*n* = 2) or medialization thyroplasty (*n* = 2)] and in two procedures the patients (*n* = 2) were lost to follow-up. Out of 18 procedures VHI-improvement at 12 months could be calculated; 15 procedures had a clinically relevant improvement (≥ 10 points) in the VHI-30. Three out of 18 had a non-relevant improvement (< 10 points). We, therefore, estimate the failure rate within 12 months for individual procedures to be somewhere between 16.7% (4/24 procedures) if considering only procedures requiring revision as failures and 37.5% (9/24 procedures) if considering procedures requiring revision and procedures without clinically relevant VHI improvement as failures and hypothesizing that the two procedures lost to follow-up were also failures.

## Discussion

In this study, we evaluated the voice outcome data after bilateral VFI of autologous fat in patients with vocal fold atrophy with or without sulcus. We found a statistically significant and clinically relevant improvement in the VHI-30 for the study group as a whole. This improvement was consistent in both patient groups (atrophy alone and atrophy with sulcus). Therefore, our results show that bilateral VFI with autologous fat is a beneficial treatment for patients with vocal fold atrophy with or without sulcus. Additionally, our results suggest that it is equally beneficial in both these patient groups. Although our results demonstrated voice improvement, there was no normalization of the voice and/or voice use based on the 12-month postoperative VHI-30 score remaining above 15 points, which indicates an elevated patient-perceived voice-related handicap.

The improvement in the VHI is supported by a statistically significant improvement in the dynamic range. Although there are no clear definitions as to what constitutes a clinically meaningful improvement in this parameter, we believe that this improvement in volume range from 32.3 to 41.1 dB is most likely meaningful to the patient, taking into account that range of 46 dB is considered acceptable for a normal voice [11]. Functionally, this reflects the likelihood that patients will notice that less effort is required to produce a softer or louder voice. Effort, and not the quality of the voice, is often the main complaint of patients with atrophy with or without sulcus and the primary aim when performing these procedures is most often to improve their voice function.

Several studies have shown a beneficial effect of autologous fat injection on glottic insufficiency from both paralytic and non-paralytic causes [12–20]. Autologous fat injection is known to be safe [21], but the long-term (> 1 year) benefits are less consistent [12, 19, 22, 23]. The effectiveness of autologous fat injection in patients with non-paralytic glottic insufficiency caused by atrophy with or without sulcus has been studied to a lesser extent, with generally positive subjective results [19, 22–24]. Chen et al. reported excellent results in 62.5% of their patients based on subjective patient ratings, together with an overall significant improvement in the perceptual rating of grade, roughness, and breathiness, and videolaryngostroboscopic rating [22]. Although Chen and others suggest that the results of fat injection are better in patients with atrophy alone than atrophy with sulcus [14, 22], this was not confirmed in our study. Recently, Dominguez et al. reported good results for patients with vocal atrophy and/or paresis treated with fat injection or bilateral medialization thyroplasty [19]. However, they found that only the thyroplasty group maintained this effect during the whole follow-up period (19 months for fat injection, 16.3 months for thyroplasty). Although the reabsorption of fat is a well-acknowledged downside of the procedure, which we anticipated, we did not see this when looking at our overall VHI-30 scores at 12 months. However, failure rate in the first 12 postoperative months in this study was estimated to be between 17 and 37.5%. A longer follow-up is required to more definitely establish the life span of fat injections in our population. Possible explanations for the discrepancies in the literature on this topic can be the amount of overcorrection or the technique of fat preservation used and the timing of post-operative measurements. It is important to establish firmly if long-term results do indeed favor thyroplasty over VFI as medialization technique.

To determine the optimal treatment for sulcus, we compared our results from bilateral VFI with autologous fat with those from other forms of sulcus surgery. The main alterative is microphonosurgery to free the epithelium and many different techniques have been described, mostly retrospectively and with varying effects.

Stuut et al. found no improvement in VHI (pre-operative 48.8, postoperative 47.1) in their patients with sulcus glottidis (*n* = 17) using solely the epithelium-freeing technique as described by Bouchayer [25]. Therefore, it has been suggested by them and by others to not only dissect the sulcus, but to restore the layered structure of the vocal folds to improve treatment outcome. Positive results have been achieved using transplantation of autologous temporalis fascia into the vocal fold (ATFV). In a study showing long-term results of ATFV in 21 patients, the VHI-10 decreased by 8.35 points after 6 months and 13.53 points after 44 months. These improvements were both statistically (*p* < 0.001) and clinically relevant (≥ 5 points on the VHI-10 [26]) [27]. However, the mean VHI-10 at last follow-up was still just above 11 points, which is considered abnormal [26]. A recent cohort of ten patients with sulcus vocalis (*n* = 6) and vocal fold scar (*n* = 4) confirmed the beneficial effect of ATFV with significant improvements in the VHI-10, perceptual grading, as well as aerodynamic (MPT, *s*/*z* ratio) and stroboscopic findings 6 months after the operation [28]. There are also studies that combine the excision of the sulcus with medialization of the vocal fold [29–31]. Yilmaz reported on 44 patients with sulcus who underwent excising of the sulcus followed by injection medialization with hyaluronic acid or calcium hydroxyapatite (*n *= 42) or bilateral type 1 thyroplasty (*n* = 2). The VHI-30 improved significantly (90.5–39.1 points) at a mean follow-up of 30 months. There was also significant improvement in the grade, roughness and breathiness of the GRBAS score, glottal closure and mucosal wave amplitude of the stroboscopic findings, and in most of the aerodynamic and acoustic parameters [29]. Miaskiewicz recently showed an improvement in the VHI-30 of 10.2 points (preoperative 44.36 versus postoperative 34.12) at 12 months after combined sulcus excision and VFI, using a short-acting filler in 29 cases, a long-acting filler in 2, a combination of fillers in 2, and no filler in 3 cases [30].

The use of angiolytic lasers for sulcus vocalis is a relatively new treatment option conceptually based on using selective photothermolysis to soften scar tissue [32–34]. A recently published series of 79 patients showed an improvement in the VHI-30 of 19.76 points, to a score close to 40 points after 6 months, as well as a significant improvement in the GRBAS by 1.07 points. Several objective voice parameters (noise to harmonic ratio, jitter, shimmer, MPT) also improved significantly in this study [34].

Despite their different surgical approaches to treat sulcus, the studies above show statistically significant and clinically relevant improvements in VHI scores, although these are still above normal [27, 29, 30, 34]. It is important to note that these results are in line with our results from treating sulcus with autologous fat injection only. Therefore, at the moment these techniques can be considered equal as to short-term (12 months) treatment effect. This is supported by the findings of Welham et al. in their prospective trial comparing three types of surgery for sulcus: type I thyroplasty, VFI with synthetic filler, and graft implantation. They concluded that no single treatment option is completely successful and there is no evidence-based decision algorithm available to identify the optimal treatment for an individual patient with sulcus [35]. Therefore, at this stage, our findings support the recommendation in the ELS consensus report to start with the least invasive technique for sulcus [4], i.e., VFI. We emphasize the importance of larger prospective studies comparing the different surgical techniques to establish optimal treatment algorithms for these patients.

Our study has some limitations. Our patient population was relatively small, although it was one of the larger study cohorts on this subject. We advocate a prospective study with a larger cohort. Also, our follow-up at this time was limited to 12 months. As stated above, a longer follow-up would better establish the life span of fat injections in our population.

## Conclusion

This study shows a statistically significant and clinically relevant subjective improvement up to 12 months after VFI with autologous fat in patients with vocal fold atrophy with or without sulcus. This improvement was reflected in the dynamic capabilities of the patients’ voices. The degree of improvement was similar in patients with or without sulcus. This indicates that bilateral VFI with autologous fat is a beneficial treatment option not only for patients with atrophy but also for patients with sulcus. Comparing our results with those from other forms of sulcus surgery in the literature confirms this finding; although we emphasize the need for further prospective studies comparing the short- and long-term effects of different techniques.
